# Veterinary Lectures in Agricultural Districts

**Published:** 1899-04

**Authors:** 


VETERINARY LECTURES IN AGRICULTURAL DISTRICTS. The German Agricultural Society in the political district of Könignihof (Bohemia) has lately held meetings in the communities of Rennzähn and Hermanitz. At these District Veterinarian Fischel has given lectures. In Rennzähn he handled the question, "What significance for the development of cattle-breeding will the epidemic law and the extinction of cattle pests have?" In Hermanitz he spoke on the nature and significance of splenic fever. Both meetings were well attended, and the farmers present expressed hearty approbation of the valuable suggestions. At the first meeting the following resolution was unanimously adopted: "The German Agricultural Society of the District of Königinhof regards it as absolutely necessary in the interests of pest-extinction, increase of cattle exports, and of agriculture in general, that the practice of veterinary medicine be granted only to qualified veterinarians, and the government is requested as soon as possible to undertake the regulation of veterinary practice with appropriate laws."—*Idem.*

